# Complicated anorectal sepsis: Validation of scoring system for predicting anorectal sepsis severity

**DOI:** 10.1097/MD.0000000000037377

**Published:** 2024-03-01

**Authors:** Amro Elhadidi, Mohammed Al-Katary, Mohamed Abdelhalim, Ahmed Negm, Ashraf Shouma

**Affiliations:** a Department of Surgery, Mansoura University, Mansoura, Egypt.

**Keywords:** anal fistula, anorectal sepsis, perianal abscess, scoring system, sepsis, validation score

## Abstract

Anorectal sepsis is a common and potentially serious medical condition characterized by infection and inflammation of the anal canal and surrounding tissues. However, the lack of standardized and comprehensive scoring systems specifically tailored for predicting the severity of anorectal sepsis poses challenges in clinical practice. This study aimed to develop and validate a scoring system for predicting the severity of anorectal sepsis by incorporating relevant patient factors. A retrospective cohort study was conducted at Mansoura University Hospital, a tertiary care center, over a period of 5 years. The study population consisted of 330 patients diagnosed with anorectal sepsis during the study period. A scoring system was developed using multiple variables, with each variable assigned a specific score based on its clinical significance and weight in predicting disease severity. The developed scoring system’s predictive performance was evaluated using receiver operating characteristic (ROC) analysis, calculating the area under the ROC curve to assess discriminative ability. Descriptive statistics were used to summarize the demographic and clinical characteristics of the study population. Chi-square tests or *t* tests were performed to assess differences between non-severe and severe anal sepsis groups. The scoring system consisted of 12 variables, with a maximum total score of 18. The logistic regression analysis revealed significant associations between localized swelling, presentation within 72 hours, multiple drainage sessions, and severe anorectal sepsis. The ROC analysis showed an area under the curve of 0.85, indicating good discriminative ability of the scoring system. The scoring system was developed and validated in a single center, which may limit its generalizability to other settings. The scoring system demonstrated good predictive performance and can be a valuable tool for clinicians in assessing disease severity, guiding treatment decisions, and identifying high-risk patients.

## 1. Introduction

Anorectal sepsis is a common and potentially serious medical condition characterized by infection and inflammation of the anal canal, rectum, and surrounding tissues.^[1]^ Untreated cases can cause serious issues, such as abscess formation, fistula development, and septic shock.^[2]^ A prompt and accurate assessment of disease severity is crucial for guiding appropriate treatment strategies and predicting patient outcomes. However, there is a lack of standardized and comprehensive scoring systems specifically tailored for predicting the severity of anorectal sepsis.^[3]^ Existing literature highlights the importance of accurate assessment and prediction of disease severity in anorectal sepsis but falls short in providing a practical and validated scoring system.^[4]^ To address this gap, there is a need to develop a reliable and validated scoring system that considers relevant patient factors associated with the severity of anorectal sepsis.

Several studies have identified factors associated with such severity of, including existing comorbidities, age, inflammatory markers, and prolonged hospital admissions.^[5,6]^ However, there is no consensus on how to incorporate these factors into a comprehensive scoring system. This lack of standardization makes it difficult to accurately assess and predict disease severity, which can lead to variability in treatment approaches and potentially worse patient outcomes.

The development and validation of a scoring system for predicting the severity of anal sepsis is an important step towards improving the management of this condition. The scoring system used in this study considers several clinical and laboratory parameters known to be associated with the severity of anorectal sepsis. By using a comprehensive approach, the scoring system can provide a more accurate prediction of the severity of anorectal sepsis compared to other methods that rely on a single parameter.

To fill this existing gap in the literature, this study aims to introduce and validate a simple and comprehensive scoring system for predicting the severity of anorectal sepsis. The scoring system will incorporate various patient factors identified in the literature.

## 2. Materials and methods

### 2.1. Study design and population

This retrospective cohort study was conducted at Mansoura University Hospital, a tertiary care center, over a period of 5 years, from 2019 to 2023. The study population consisted of patients diagnosed with anorectal sepsis during the study period. In our study, we specifically targeted anal sepsis, with a particular focus on perianal sepsis and abscess formation. The inclusion criteria were thus patients diagnosed with these conditions. However, we excluded several conditions from our research scope to maintain the specificity of our study. These excluded conditions encompassed decubitus ulcers, bed sores, and any complications that arose post-proctologic surgeries such as post abdominoperineal wound infection, In our study, the term “anorectal sepsis” refers to a variety of perianal and perirectal infections that do not include severe necrotizing forms. Patients with incomplete medical records or missing data were excluded from the analysis.

### 2.2. Data collection

A thorough review of electronic medical records was performed to collect relevant data for each patient. The data collection process included demographic information, presenting symptoms, associated comorbidities, laboratory investigations (including C-reactive protein (CRP), white blood cell (WBC) count, and serum lactate levels), need for multiple drainage sessions, Intensive care unit (ICU) admission, and anal sphincter damage. The data were extracted by trained research personnel and entered into a secure database.

### 2.3. Development of the scoring system

**Figure 1.**
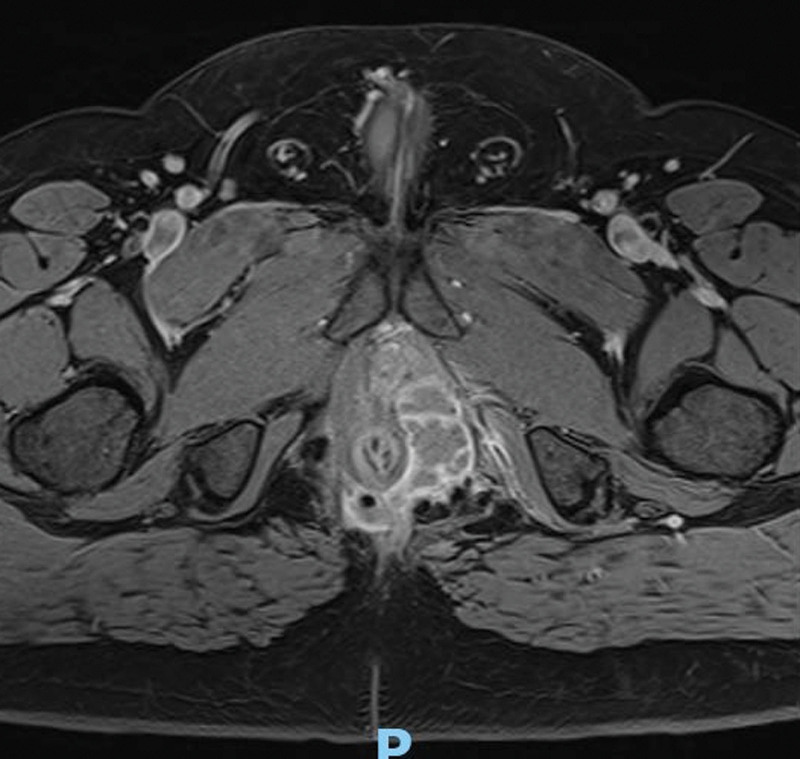
MRI showed localized large deep-seated anorectal sepsis. MRI = magnetic resonance imaging.

**Figure 2.**
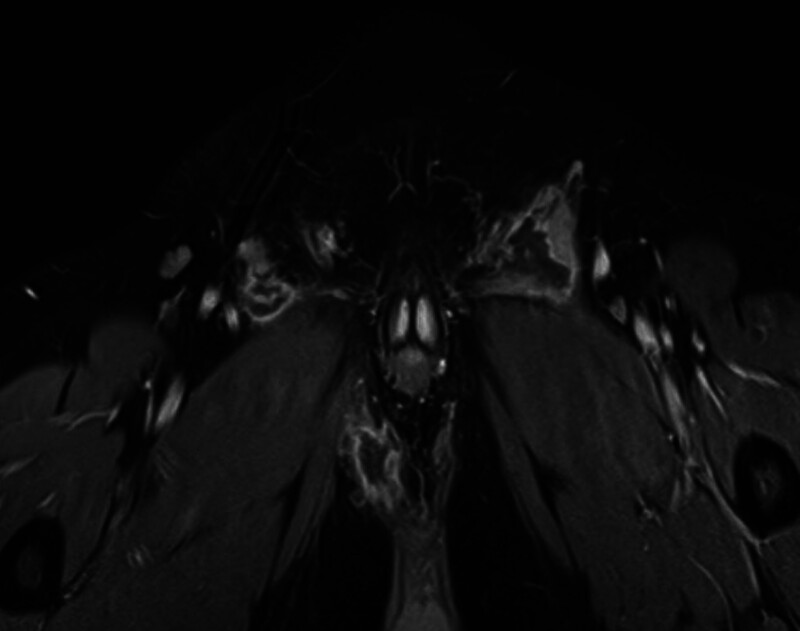
MRI showed diffuse multiloculate large deep-seated anorectal sepsis. MRI = magnetic resonance imaging.

**Figure 3.**
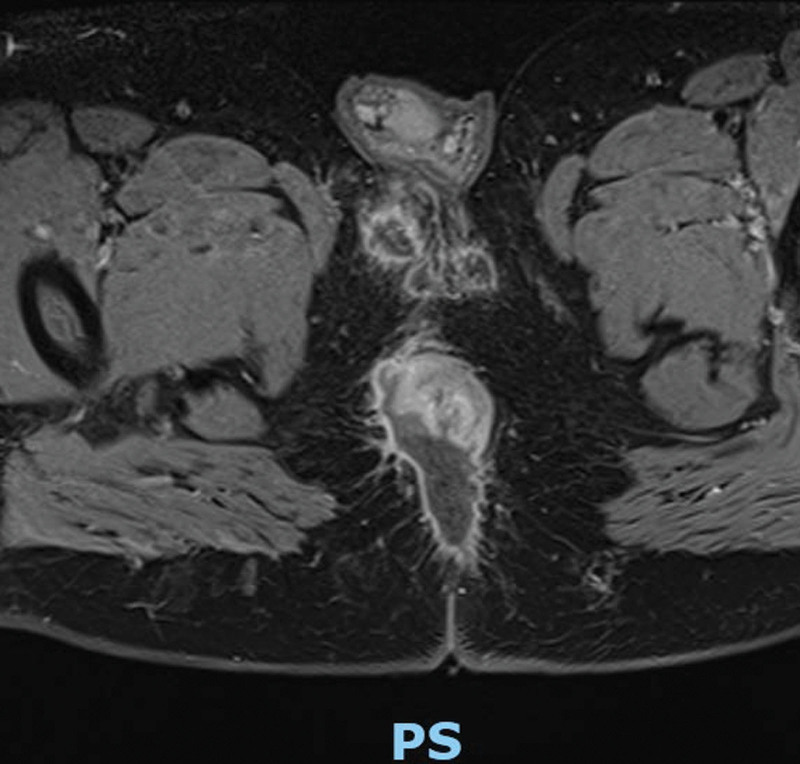
MRI showed anal sphincteric damage due to severe anorectal sepsis and multiple surgical drainage. MRI = magnetic resonance imaging.

The “Complicated Anorectal Sepsis Severity Score” (CASSS) was developed based on multiple variables. Each variable included in the scoring system was assigned a specific score based on its clinical significance and weight in predicting disease severity^[7]^ (Table 1). Perianal swelling in such patients is typically defined and classified into 1 of 2 types in clinical practice: Localized, well-defined inflammatory phlegmon or abscess: This category includes abscess cavities that are well-circumscribed and can be perianal, inter-sphincteric, or submucous abscesses in which the size of the abscess is a well-known parameter (Fig. 1). Diffuse swelling: Unlike the previously mentioned localized abscess, this category refers to diffuse, non-well-circumscribed sepsis that does not adhere to a single defined anal plane (Fig. 2). It is important to note that evaluating associated sphincteric fistulae in such patients can be difficult, and postoperative follow-up with specific radiological studies such as magnetic resonance imaging may reveal fistula formation with management tailored to its exact extension. However, this was not the focus of our investigation. In the current iteration of our scoring system, we have grouped all preoperative comorbidities into a single category, and they are scored equally. While we assumed that not all comorbidities carry the same clinical weight, we chose this simplified approach to maintain the scoring system’s ease of use and practicality in clinical settings. Regarding the variable “anal sphincter damage,” this term refers to patients who do not have an existing stoma at the time of presentation but have a clinical condition that can necessitate the creation of a stoma or sphincteric damage either by sepsis or operative drainages. Patients with sphincteric damage were evaluated through history, physical examination, digital rectal examination under anesthesia, and radiological examination (endoanal ultrasound and magnetic resonance imaging or computed tomography imaging) (Fig. 3). Our research focuses on anorectal sepsis as it relates to perianal sepsis and abscess formation. Other conditions such as decubitus ulcers, bed sores, or any postoperative complications after proctologic surgeries such as post abdominoperineal wound infection, dehiscence, and sepsis were excluded in the context of our research methodology.

**Table 1:** Development of the scoring system.

Variables	Scoring
1. Age (≥60 yr)	1
2. Diffuse perianal swelling	1
3. Constitutional symptoms (fever)	1
4. Time of presentation ≥ 72 h	2
5. Any preoperative comorbidities	1
6. WBCs ≥ 18,000	1
7. Elevated c-reactive protein (CRP)	1
8. High serum lactate	2
9. Multiple drainages	2
10. ICU admission ≥ 2 days	2
11. Anal sphincters damage	3
12. Stoma creation	1
**Total score**	**18**

CRP = C-reactive protein, ICU = intensive care unit, WBC = white blood cell.

### 2.4. Statistical analysis

Descriptive statistics were used to summarize the demographic and clinical characteristics of the study population. Continuous variables were presented as means with standard deviations or medians with interquartile ranges, depending on their distribution. Categorical variables were presented as frequencies and percentages. To assess the differences between non-severe and severe anal sepsis groups, the groups were classified using a comprehensive scoring system we developed called the CASSS, which considers a variety of clinical parameters and severity indicators. Chi-square tests or *t* tests were performed for categorical and continuous variables, respectively. Logistic regression analysis was conducted to identify significant parameters associated with severe anorectal sepsis. Variables with a *P* value < .05 in the univariate analysis were included in the multivariate logistic regression model. The predictive performance of the developed scoring system was evaluated using receiver operating characteristic (ROC) analysis.^[8]^ The area under the ROC curve was calculated to assess the discriminative ability of the scoring system in predicting severe anorectal sepsis. Sensitivity, specificity, positive predictive value, and negative predictive value were also determined at various cutoff points to identify the optimal threshold for clinical decision-making. All statistical analyses were performed using Stata 16.1. Significance levels were set at *P* < .05. In our study, we conducted a power analysis using Stata. Sample size: Given the study parameters of 0.05 alpha, 0.80 desired power, and a true effect size (delta) of 0.30, the current sample size of 330 participants provides adequate statistical power (80%) to detect the expected difference. The command used in Stata for this calculation was power two means.

### 2.5. Ethical considerations

The Ethics Committee of Mansoura University Hospitals granted ethical approval for this study. All procedures in this study were carried out in accordance with the institutional and/or national research committee’s ethical standards, as well as the 1964 Helsinki Declaration and its subsequent amendments or comparable ethical standards.

## 3. Results

### 3.1. Study population

A total of 330 patients diagnosed with anorectal sepsis were included in the study. Table 2 presents the distribution of variables among the severe and non-severe groups, along with the corresponding *P* values. A univariate analysis was performed to assess the factors associated with the severity of anorectal sepsis. Gender did not show a significant association with severity (*P* = .285). Body mass index (BMI) demonstrated a statistically significant difference between the 2 groups (*P* = .0049), with a higher BMI observed in the non-severe group. CRP levels and lactate levels were significantly associated with severity (both *P* < .001), with higher proportions of positive CRP and elevated lactate levels in the severe group. Presentation > 72 hours, WBC counts (>18,000), mortality, anal sphincter damage, ICU admission, the need for multiple drainages, the presence of a stoma, the presence of fever, comorbidities, and diffuse swelling were all strongly associated with severity (all *P* < .001). These results indicate that BMI, CRP, lactate levels, presentation time, WBC counts, mortality, anal sphincter damage, ICU admission, the need for multiple drainages, the presence of a stoma, the presence of fever, comorbidities, and diffuse swelling are important factors in determining the severity of anorectal sepsis.

**Table 2:** Baseline characteristics of participants by group.

Gender	Severe, N = 30	Non-severe, N = 300	*P* value
Male	26 (86.67)	235 (78.33)	.285
Female	4 (13.33)	65 (21.67)	
**BMI**	29 ± 4.9	31.5 ± 5	**.0049**
**CRP**			
Positive	21 (70.00)	0	**<.001**
Negative	9 (30.00)	300 (100.00)	
**Lactate**			
High	19 (63.33)	0 (0.00)	**<.001**
Normal	11 (36.67)	300 (100.00)	
**Presentation within 72 h**			
Less 72	7 (23.33)	291 (97.00)	**<.001**
More 72	23 (76.67)	9 (3.00)	
**WBCs**			
<18,000	7 (23.33)	288 (96.00)	**<.001**
>18,000	23 (76.67)	12 (4.00)	
**Mortality**			
Yes	4 (13.3)	0	**<.001**
No	26 (86.7)	300 (100)	
**Anal sphincters damage**			
Yes	3 (10)	0	**<.001**
No	27 (90)	300 (100)	
**ICU**			
No	22 (73.3)	0	**<.001**
Yes	8 (26.7)	300 (100)	
**Multiple drainages**			
Yes	24 (80)	5 (1.7)	**<.001**
No	6 (20)	295 (98.3)	
**Stoma**			
Yes	8 (26.7)	0	
No	22 (73.3)	300 (100)	
**Fever**			
Yes	24 (80)	18 (6)	**<.001**
No	6 (20)	282 (94)	
**Comorbidities**			
Yes	26 (86.7)	24 (8)	**<.001**
No	4 (13.3)	276 (92)	
**Diffuse swelling**			
Yes	25 (83.3)	9 (3)	**<.001**
No	5 (16.7)	291 (97)	

BMI = body mass index, CRP = C-reactive protein, ICU = intensive care unit, WBCs = white blood cells.

**Figure 4.**
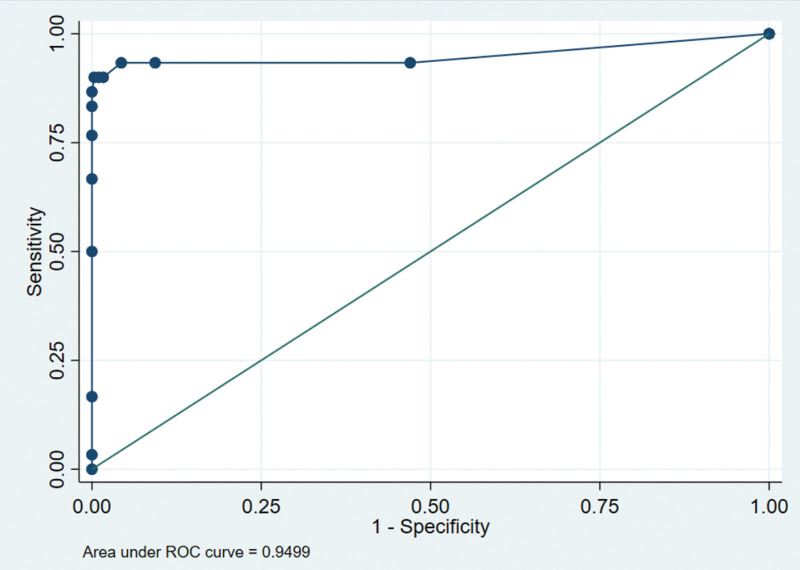
Receiver operating characteristic (ROC) curve for predicting the severity of anal sepsis.

The ROC analysis was conducted to evaluate the predictive performance of the scoring system for the severity of anorectal sepsis (Fig. 4). The area under the ROC curve was found to be 0.9576. Table 3 demonstrates patients with a score of 8 or higher are correctly classified as having a severe form of anorectal sepsis with a high level of accuracy. The scoring system shows a strong ability to identify individuals who are at a higher risk of experiencing severe complications related to anorectal sepsis. Therefore, using a cutoff point of ≥8 can be considered effective in distinguishing between patients with different levels of severity and guiding appropriate treatment decisions. Table 4 presents the logistic regression results for the predictors and the outcome variable (GROUP) in patients with anorectal sepsis. The findings indicate that localized swelling, presentation within 72 hours, and no multiple drainage are significantly associated with non-severe anorectal sepsis. Diffuse swelling showed a significant positive association with the severity of anorectal sepsis (*P* = .008). Late presentation (>72 hours) was also positively associated with the severity of anorectal sepsis (*P* = .041). Similarly, multiple drainages showed a positive association with the severity of anorectal sepsis (*P* = .007). Patients requiring multiple drainage procedures had higher odds of belonging to the severe group.

**Table 3:** Performance of the scoring system at different cutoff points for predicting severe anal sepsis.

Cutoff point	Sensitivity	Specificity	Classified
≥2	100.00%	0.90%	9.09%
≥3	93.33%	53. 00%	56.67%
≥4	93.33%	90.67%	90.91%
≥5	93.33%	95.67%	95.45%
≥7	90.00%	98.33%	97.58%
≥8	90.00%	99.00%	98.18%
≥9	90.00%	99.67%	98.79%
≥10	86.67%	100.90%	98.79%
≥11	83.33%	100.00%	98.48%
≥12	76.67%	100.00%	97.88%
≥13	66.67%	100.00%	96.97%
≥14	50.00%	100.00%	95.45%
≥15	16.67%	100.00%	92.42%
≥16	3.33%	100.00%	91.21%
>16	0.00%	100.00%	90.91%

**Table 4:** Logistic regression analysis of factors associated with severity of anal sepsis.

Group	Beta coefficient	*P* value	LCI	UCI	Sig
Localized swelling	.1	.008	.018	.541	***
Constitutional symptoms—No	.24	.102	.044	1.326	
Presentation < 72 h	.17	.041	.03	.932	**
Multiple drainage—No	.08	.007	.014	.501	***
Constant	290.232	0	19.656	4285.334	***

***P < .01, **P < .05, *P < .1.

## 4. Discussion

Anorectal sepsis is a complicated condition with a wide range of disease severity and clinical outcomes. It can manifest in a variety of ways, ranging from minor infections that can be treated conservatively to severe cases that necessitate surgical intervention. Anorectal sepsis has the potential to cause septic shock and even death in the most severe cases. The presentation and degree of inflammation associated with anal sepsis vary greatly, highlighting its unpredictable nature.^[9,10]^ Severe anorectal sepsis may necessitate extensive surgical interventions, such as debridement and multiple drainages. Comorbidities and late-stage disease are common in these cases, with patients displaying clinical and biochemical changes indicative of severe infection. Prompt treatment is critical for these patients to avoid further complications. Based on previous research and its clinical significance, we carefully selected specific criteria to evaluate the extent of anorectal sepsis in this study. We provide detailed explanations for each criterion and its importance in predicting sepsis severity.

The mortality and morbidity rates associated with sepsis are heavily influenced by age and delayed diagnosis. These elements are crucial components of various scoring systems, including Acute Physiology and Chronic Health Evaluation II, Ranson criteria, and Charlson criteria. Furthermore, advanced age is widely acknowledged as a risk factor for increased severity of sepsis due to weakened immune responses in the elderly and the presence of comorbidities that worsen disease outcomes.^[11,12]^

Detecting widespread inflammation is indicated by the presence of diffuse perianal swelling. This suggests a higher probability of tissue involvement and indicates a more extensive inflammatory process.^[13,14]^ Unlike well-defined localized abscess collections that require simple incision and drainage, diffuse perianal swelling usually indicates a more severe and complex condition. This suggests an underlying disease process or complication, such as anorectal sepsis. For accurate diagnosis and appropriate management in these cases, various factors such as patient symptoms, history, and evaluation findings must be considered.

In the context of sepsis, fever serves as a significant indicator of infection and inflammation. It plays a crucial role in signaling an activated immune response. A prolonged or high-grade fever may suggest a more severe and widespread infection.^[15]^ In the management of sepsis, it is crucial to intervene promptly. Any delays in seeking treatment beyond 72 hours can lead to the progression of the disease, resulting in more severe tissue damage, formation of abscesses, or systemic complications. Comorbidities such as diabetes, immunosuppression, or chronic medical conditions have been unequivocally linked to an increased risk of severe sepsis. These comorbidities can impair the body’s ability to mount an effective immune response and complicate treatment options.^[16,17]^ Higher levels of lactate and WBCs are important indicators of sepsis severity. Elevated WBCs, as well as increased lactate and procalcitonin levels, have been identified as reliable markers associated with more severe cases of sepsis, providing useful information about disease severity in anal sepsis. A WBC count of 18,000 or higher is commonly indicative of infection and inflammation, implying a stronger immune response to the infection and possibly indicating a more severe episode of sepsis.^[18]^ CRP is an acute-phase reactant that elevates in response to inflammation. It is a reliable indicator of inflammatory processes, with higher CRP levels associated with more severe infections or inflammation.^[19]^ Additionally, elevated serum lactate levels may indicate tissue hypoxia, implying systemic involvement and organ dysfunction. Because this occurrence is frequently associated with severe sepsis and septic shock, high lactate levels can be used to assess the severity of sepsis.

The management of complicated and severe cases of anal sepsis often involves the need for multiple drainage and surgical procedures, which can result in higher healthcare resource usage and expenses. Additionally, these patients usually require longer stays in the intensive care unit and postoperative hospitalization periods, placing a significant burden on both patients and healthcare systems. The necessity for repeated drainage and debridement procedures is indicative of a more complex or widespread infection that requires ongoing treatment. Having multiple drains may be an indication of severe sepsis that poses difficulties in its treatment.^[20,21]^ Patients who require more than 2 days of ICU admission often require intensive care and support due to the severity of sepsis, which increases their risk of complications.^[22]^

The anal sphincters damage, whether caused by sepsis or surgical procedures, can have a substantial impact on the quality of life and long-term outcomes for patients. Individuals who suffer from this loss often display more severe instances of anal sepsis. In situations where there is significant tissue damage or dysfunction due to severe anal sepsis, the creation of a stoma is frequently required as it signifies a more advanced and intricate stage of the condition.^[4,10]^

There are several well-known severity scores in the field of sepsis that are commonly referenced, such as the Acute Physiology and Chronic Health Evaluation II and SIRS criteria. However, when it comes to accurately assessing the severity of anorectal sepsis, these scoring systems have limitations. They lack the specificity required for a thorough evaluation.

The development of a comprehensive scoring system for anorectal sepsis has the potential to improve clinical practice. By incorporating relevant patient factors into a validated scoring system, clinicians will have a standardized tool for assessing disease severity and guiding treatment decisions. This will help in clinical practice for informed decision-making for patients with anorectal sepsis. A validated scoring system specific to anal sepsis will also enable the identification of patients at high-risk for severe local and systemic complications. Early recognition of these high-risk patients can facilitate timely interventions, such as surgical drainage or antibiotic therapy, and potentially prevent the progression of disease and improve patient outcomes.

Our study was conducted on 330 patients diagnosed with anorectal sepsis and found that several factors were strongly associated with the severity of anorectal sepsis. These factors included BMI, CRP levels, lactate levels, presentation time, WBC counts, mortality, anal sphincters damage, ICU admission, the need for multiple drainages, presence of a stoma, presence of fever, comorbidities, and diffuse swelling. These findings are consistent with previous studies that have identified similar risk factors for anorectal sepsis.^[23,24]^

We also conducted ROC analysis and found that using a cutoff point of ≥8 can be effective in distinguishing between patients with different levels of severity and guiding appropriate treatment decisions. This finding is important as it suggests that a simple scoring system based on easily measurable clinical parameters can be used to identify patients at higher risk of severe anorectal sepsis and guide appropriate treatment decisions.

The CASSS aimed to address a critical need in clinical practice. Anorectal sepsis, a condition encompassing a common surgical entity, can pose challenges in terms of diagnosis and management. This lack of a standardized and validated scoring system has resulted in variations in clinical assessment and treatment approaches. The CASSS was created to provide a methodical and objective way of determining the severity of anorectal sepsis. It considers a variety of clinical parameters and comorbidities to classify patients into severity groups, which can help with clinical decision-making. Furthermore, the CASSS’s importance stems from its ability to prevent medicolegal issues. It helps ensure that patients receive appropriate care and management by providing a standardized and validated approach to assessing anal sepsis severity, lowering the risk of adverse outcomes. This is especially important in cases where anal sepsis is misdiagnosed and mistreated, resulting in significant morbidity and mortality if not properly evaluated and treated.

One of the strengths of the scoring system is its simplicity and ease of use. The scoring system can be easily calculated using readily available clinical and laboratory parameters, which makes it practical for use in a clinical setting. Given the lack of established scoring systems for anal sepsis, there is a need for research and development in this area. Developing a validated scoring system that incorporates relevant clinical and laboratory parameters specific to anorectal sepsis could greatly contribute to standardized assessment, risk stratification, and management of this condition.

However, there are some limitations to the scoring system that should be considered. The scoring system was developed and validated in a single center, which may limit its generalizability to other settings. Additionally, the scoring system may not be able to predict the severity of anal sepsis in all cases, as there may be other factors that contribute to the severity of the condition.

Future research should focus on further validating the scoring system in other settings and populations. Additionally, the scoring system could be further refined by incorporating additional parameters or by using machine learning algorithms to improve its accuracy.

## 5. Conclusion

Validating a scoring system for anal sepsis is crucial for predicting patient outcomes and optimizing management plans. The proposed scoring system incorporates various patient factors and has shown promising results in predicting mortality and morbidity. By utilizing this validated scoring system, healthcare professionals can effectively assess disease severity, identify high-risk patients, and tailor appropriate treatment strategies to improve patient outcomes. Further research and implementation of this scoring system are warranted to enhance the care and management of anorectal sepsis.

## Author contributions

**Conceptualization:** Amro Elhadidi, Mohammed Al-Katary.

**Data curation:** Amro Elhadidi, Mohammed Al-Katary, Mohamed Abdelhalim.

**Formal analysis:** Amro Elhadidi, Mohammed Al-Katary, Mohamed Abdelhalim.

**Funding acquisition:** Amro Elhadidi.

**Investigation:** Amro Elhadidi, Mohammed Al-Katary, Mohamed Abdelhalim, Ahmed Negm, Ashraf Shouma.

**Methodology:** Amro Elhadidi, Mohammed Al-Katary, Mohamed Abdelhalim, Ahmed Negm, Ashraf Shouma.

**Project administration:** Amro Elhadidi.

**Resources:** Amro Elhadidi, Ahmed Negm, Ashraf Shouma.

**Software:** Amro Elhadidi.

**Supervision:** Amro Elhadidi, Mohammed Al-Katary, Ahmed Negm, Ashraf Shouma.

**Validation:** Amro Elhadidi, Mohammed Al-Katary, Mohamed Abdelhalim.

**Visualization:** Amro Elhadidi.

**Writing – original draft:** Amro Elhadidi, Mohammed Al-Katary, Mohamed Abdelhalim.

**Writing – review & editing:** Amro Elhadidi, Mohammed Al-Katary, Mohamed Abdelhalim.
